# Depression level, nutritional status, and dietary nutrient intake of the older adult at the community level in a selected area of Bangladesh

**DOI:** 10.1016/j.heliyon.2023.e18199

**Published:** 2023-07-13

**Authors:** Tasmia Tasnim, Md Zafar As Sadiq, Kazi Muhammad Rezaul Karim

**Affiliations:** a Department of Nutrition and Food Engineering, Daffodil International University, Daffodil Smart City, Birulia, 1216, Savar, Dhaka, Bangladesh; b Institute of Nutrition and Food Science, University of Dhaka, Dhaka, 1000, Bangladesh

**Keywords:** Malnutrition, Depression, Older adult, Energy, Nutrient, Bangladesh

## Abstract

Malnutrition is associated with higher rates of morbidity and death in the older population. Depression or mental health is a major component of older adult malnutrition. The aim of the study was to measure the level of malnutrition and depression in older adults, as well as their correlated factors, such as dietary energy and nutrient consumption. A cross-sectional study was conducted among 108 older individuals living in two areas of Faridpur, Bangladesh. The Mini Nutritional Assessment-Short Form (MNA-SF), Geriatric Depression (GD) Scale, and 24-h dietary recall were used to measure the nutritional status, depression level, and dietary nutrients, respectively. A total of 20.4% and 55.6% were malnourished or at risk of malnutrition, respectively. Around 81.5% of the study subjects exhibited a different degree of depression and 9.3% were identified as having severe depression. There was a significant inverse association between the MNA-SF score and the GD score (r = −0.684, p=<0.001). The average energy and protein consumption was 1387 kcal and 45.52 g, respectively; and energy and protein intake were significantly lower in the depressed group (1353 Kcal, 43.8 g) than in the non-depressed group (1530 Kcal, 52.4 g). An extremely low energy consumption (<20 kcal/kg body weight/day) was noted in 27.1% of the older adults. None of the participants in this study were able to meet the requirements for dietary fiber, calcium, vitamin B6, folate, vitamin D, and vitamin E. Specific nutrition-related intervention programs as well as social and familial support are recommended to improve the nutritional status of older adults.

## 1 Introduction

Over the next three decades, the number of people nearly 65 years old, who today make up 12% of the world's population, will increase to over 1.5 billion [1]. By 2050, 42.2 million older adults are anticipated to reside in Bangladesh [2]. The basis of a healthy existence is good nutrition. Malnutrition is linked to greater rates of morbidity and mortality, as well as higher expenses for medical treatment and longer inpatient stays [3]. Since aging is accompanied by physiological changes such as decreased appetite, lower energy expenditure, weight loss, changes in taste, and changes in smell, incorporated with depression and loneliness, older persons are generally more prone to nutritional deficiencies [[4], [5], [6], [7]]. Additionally, inadequate nutrition in older persons is strongly linked to a decreased risk of functional capacity, decreased cognitive performance, and higher mortality [8,9]. However, limitations can also impact one's mental and/or social integrity in addition to their ability to perform basic bodily tasks. Geriatric depression is a mental illness that affects older persons, impairs quality of life, and raises the risk of suicide [10]. Depression or mental health were identified as an important factor in malnutrition for the older adult, as they alter the appetite, sleep habits, and cognitive function [6,7,11]. The mental health of older adults is one of the least important concerns in Bangladesh [12]. At the same time, both physical and mental health are essential to achieving sustainable development goals (SDG) [13].

Malnourishment is a widespread issue everywhere in the world. Up to 50% of older persons are estimated to have malnutrition, which varies greatly depending on the research population, assessment methodologies, and healthcare environment [14,15]. However, the prevalence of malnutrition in Bangladesh was reported in a few studies to be higher in nursing homes or old age homes (33.5%) compared to community or free-of-cost homes for living (25–26%) [[6], [7], [8]]. In elderly people, nutrition plays a significant role in regulating health and wellbeing. As we all know, as we get older, our capacity to chew, our ability to digest food, our appetite, and our level of physical activity decline, resulting in reduced dietary consumption [16]. It has repeatedly been noted that elderly persons frequently consume fewer calories and nutrients than required [[16], [17], [18]]. The risk of malnutrition is raised in older people because of a variety of conditions that frequently affect dietary intake. Diet quality is also an important factor in both malnutrition and depression [19]. Only a limited study has focused on geriatric malnutrition and its associated factors in Bangladesh. At the same time, no study was found on dietary energy and nutrient intake in Bangladesh among the older population. So, the purpose of the study was to measure the rate of malnutrition and mental health status, as well as the correlated factors between malnutrition and mental health, in the selected community-living older adults of Bangladesh. The study also estimates the amount of energy and nutrients consumed by the study subjects.

## 2 Methods

### 2.1 **S**tudy designs and participants

A cross-sectional study was carried out among individuals aged 65 years and above living in the home care support program at Char Kamalapur and Chauhatta village in Aliabad Union, Faridpur Sadar, Faridpur, Bangladesh. The selected study area comprises about 500 households and about 200 older adults. A total of 108 older adults were selected for this study by convenience sampling. The survey included senior citizens who resided with their families without any serious medical complications or physical disabilities.

### 2.2 Measurement of nutritional status

The Mini Nutritional Assessment-Short Form (MNA-SF), which has six questions and scores from 0 to 14, was used to measure nutritional status [20]. The MNA-SF is divided into six categories: body mass index, recent weight loss, mobility limitation, acute illness/stress, dementia or depression, and appetite or eating problems. The total MNA-SF score is 0–7, 8–11, and 12–14 indicate malnutrition, risk of malnutrition, and well-nutrition, respectively [20,21].

### 2.3 Measurement of mental health and physical function

The Geriatric Depression Scale (GDS), which has 15 questions about the quality of life in many ways, such as hopelessness, helplessness, etc., was used to measure the level of mental health or depression [22]. Out of a possible 15 scores, 0 to 4 indicated no depression, 5–8 indicated mild depression, 9–11 indicated moderate depression, and 12–15 indicated severe depression [23].

Based on a previous study [24], physical characteristics like mobility limitations and activities of daily living (ADL) were looked at.

### 2.4 Socio-demographic characteristics

A structured questionnaire was made to get the right information about the participants' demographics, socioeconomic status, and health. Socio-demographic and lifestyle factors like age, gender, marital status, smoking, religion, level of education, etc. were taken into account. Any concurrent medical condition was also inquired about.

### 2.5 Dietary nutrient intake

The amount of dietary food consumed was measured using the 24-h dietary recall. The plates, cups, and utensils were exhibited to gain the most accurate estimate of the serving size of food ingested for the dietary record. The food consumption data of these selected individuals was processed using the “Food Composition Table for Bangladesh (FCTB-2013)” to calculate the nutrient consumption [25]. Energy, protein, and dietary fiber consumption were compared with the recommended ESPEN guidelines [26]. The micronutrient consumption was compared with FAO/WHO recommendations [27]. As there is no standard guideline for the Bangladeshi population, two references with recommended guidelines (ESPEN for macronutrients and FAO/WHO for micronutrients) were used to better understand the results.

### 2.6 Statistical analysis

The statistical software SPSS 21 was used for all of the analyses. Means and standard deviations were employed to present the results of descriptive analyses (SD). One-way ANOVA was used to compare the means between the different groups, and Tukey HSD Post Hoc Test was used to measure the difference within the groups. The Chi-square test was used to determine the association between categorical data and was expressed as frequency and percentage. The probability of P < 0.05 was statistically significant for all tests.

## 3 Results

The overall malnutrition rate was 20.4%, and more than half of the elderly population (55.6%) was at risk of malnutrition (Table 1). By the Tukey HSD test, the age was significantly higher in the malnourished group than in the well nourished and at-risk malnutrition groups. Female subjects were more vulnerable to malnutrition or at greater risk of malnutrition compared to male participants (p = 0.013) (Table 1). There was a significant association between the degree of malnutrition and various parameters, such as BMI and depression score. Significantly lower BMI was recorded in the malnourished group than in the well-nourished and at-risk of malnutrition groups. A significant chi-square test confirmed the association between the degree of malnutrition and limitations on mobility, as well as limitation on ADL (Table 1). Almost one-fifth of elderly people have limitations on both mobility (19.4%) and activities of daily living (18.5%) (Table 1). No significant association was observed between education status and nutritional status (Table 1).

**Table 1:** Socio-economic characteristics of the respondent according to nutritional status.

Variable	Malnourished (n = 22)	Risk of Malnutrition (n = 60)	Well-nourished (n = 26)	*p-value
Age in years (Mean ± SD)	72.72 ± 7.28	68.15 ± 5.87	68.42 ± 5.79	0.011
BMI (Mean ± SD)	19.57 ± 3.35	23.19 ± 2.38	22.84 ± 1.60	0.004
Weight (Mean ± SD)	61.00 ± 11.95	56.25 ± 9.24	60.84 ± 9.67	0.055
GDS (point) (Mean ± SD)	9.91 ± 2.48	7.21 ± 1.67	4.53 ± 1.60	<0.001
Sex	F**requency (%)**			**p value
Male	8 (15.4)	25 (48.1)	19 (36.5)	ƛ^2^ = 8.70, p = 0.013
Female	14 (25.0)	35 (62.5)	7 (12.5)	
Both	22 (20.4)	60 (55.6)	26 (24.1)	
**Physical Function**				
Limitation on mobility: Yes	15 (68.2)	6 (10)	0	ƛ^2^ = 43.05, p=<0.001
NO	7 (31.8)	54 (90)	26 (100)	
Limitation on ADL: Yes	14 (63.6)	6 (10)	0 (0)	ƛ^2^ = 38.47, p=<0.001
NO	8 (36.4)	54 (90)	26 (100)	
**Depression:** Yes	21 (95.5)	59 (98.3)	8 (30.8)	ƛ^2^ = 58.45, p=<0.001
No	1 (4.5)	1 (1.7)	18 (69.2)	
Family Income/Month				
Up to 7000 Tk	14 (63.8)	28 (46.7)	14 (53.8)	ƛ^2^ = 1.91, p = 0.385
More than 7000 Tk	8 (36.4)	32 (53.3)	12 (46.2)	
Education Qualification				
Illiterate	13 (59.1)	39 (65.0)	13 (50.0)	ƛ^2^ = 4.14, p = 0.388
Primary	5 (22.7)	15 (25.0)	6 (23.1)	
Secondary to Higher	4 18.2)	6 (10.0)	7 (26.9)	

* = One-Way ANOVA and Tukey HSD test Post Hoc Test; ** = Chi square test; BMI, Body Mass Index; GDS, Geriatric Depression score; ADL, Activities of daily living.

According to the Geriatric Depression Scale-Short Form (GDS-SF), most of the older adults (81.5%) showed different degrees of depression symptoms. This study found that 18.5% of participants had no depression, 54.5% had mild depression, 17.6% had moderate depression, and 9.3% had severe depression (Table 2). The proportion of moderate and severe depression was higher in females (25 and 10.7%) than in males (9.6% and 7.7%) (Table 2). The malnourished older adults had a markedly higher average GDS score (9.91 ± 2.48) compared to the subjects with ideal nutritional status (4.53 ± 1.60) (Table 1). This highest GDS score was significantly higher from both the well-nourished and at risk of malnutrition groups. A significant association between nutritional status and degree of depression was also observed in the chi-square test (Table 2).

**Table 2:** Factors Associated with depression level.

Variable	Normal/no depression (n = 20)	Mild depression (n = 59)	Moderate depression (n = 19)	Severe depression (n = 10)	*p value
Age in years (Mean ± SD)	69.3 ± 7.81	68.74 ± 6.25	68.11 ± 4.93	73.2 ± 5.65	0.188
BMI (Mean ± SD)	21.98 ± 2.78	23.51 ± 2.44	22.17 ± 1.93	20.66 ± 2.89	0.064
Weight (Mean ± SD)	59.45 ± 10.5	58.10 ± 9.92	58.25 ± 9.05	57.5 ± 12.78	0.952
**Sex**	F**requency (%)**				**p value
Male	13 (25%)	30 (57.7)	5 (9.6)	4 (7.7)	p = 0.096
Female	7 (12.5)	29 (51.8)	14 (25)	6 (10.7)	
Both	20 (18.5)	59 (54.6)	19 (17.6)	10 (9.3)	
**Nutritional status**					
Malnourished	1 (4.5)	3 (13.6)	10 (45.5%)	8 (36.4%)	ƛ^2^ = 100.8, p=<0.001
At risk of malnutrition	1 (1.7)	48 (80.0%)	9 (15.0%)	2 (3.3%)	
Well-nourished	18 (69.2%)	8 (30.8)	0	0	
**Physical function**					
Limitation on mobility: Yes	2 (10%)	6 (10.2%)	6 (31.6%)	7 (70%)	ƛ^2^ = 22.48, p=<0.001
NO	18 (90%)	53 (89.8%)	13 (68.4%)	3 (30%)	
Limitation on ADL: Yes	2 (10%)	6 (10.2%)	6 (31.6%)	6 (60)	ƛ^2^ = 17.24, p=<0.001
No	18 (90%)	53 (89.8%)	13 (68.4%)	4 (40%)	

* = One-Way ANOVA and Tukey HSD test Post Hoc Test; ** = Chi square test.

Just like with nutrition, there was a strong link between depression level and limitations in mobility and ADL (Table 2). People who were severely depressed were more likely to have trouble with physical function, both in terms of mobility and ADL.

Table 3 compared the energy and nutrient consumption levels of study subjects in different depression status groups. The average energy and protein consumption were 1387 kcal and 45.52 g, respectively. Daily energy, protein, fat, zinc, vitamin B1, and vitamin E intake were significantly lower in depressed older adults than in non-depressed individuals (Table 3). About 80% of older participants could not meet their desire for dietary energy of 30 kcal/BWkg/day, and about 27% of older adults consumed 20 kcal/kg body weight per day (Table 3). About seventy-six percent of the study subjects could not fulfill their protein requirement (1.0 g/kg body weight per day), and 57.9% consumed 0.8 g of protein per kg body weight per day (Table 3). None of the participants in this study were able to meet the requirements for dietary fiber, calcium, vitamin B6, folate, vitamin D, and vitamin E (Table 3).

**Table 3:** Energy and Nutrients consumption by the depression status, and comparison to RDA.

Nutrients	Normal/No depression (Mean ± SD)	Any kind of Depression (Mean ± SD)	*p-value	Average (Mean ± SD)	< RDA (% frequency)	<50% of RDA (% frequency)
Energy (Kcal)	1530 ± 396	1353 ± 273	0.019	1387 ± 305	80.4^a^	27.1^b^
Protein (g)	52.4 ± 19.8	43.9 ± 12.8	0.019	45.52 ± 14.64	76.6^c^	57.9^d^
Carbohydrate (g)	290.6 ± 67.4	262.9 ± 548	0.054	268.08 ± 58.1	–	–
Fat (g)	30.2 ± 10.0	24.4 ± 7	0.003	25.52 ± 7.9	–	–
Dietary Fiber (g)	16.6 ± 3.7	15.8 ± 3.4	0.393	16.03 ± 3.5	100	14.0
Calcium (mg)	315.3 ± 212	260.7 ± 186	0.253	271 ± 191	100	93.5
Magnesium (mg)	258.7 ± 78	236.0 ± 3	0.232	240.3 ± 59	36.4	0
Iron (mg)	7.5 ± 2.5	7.1 ± 2.1	0.496	7.18 ± 2.2	95.3	30.8
Zinc (mg)	7.5 ± 2.6	6.5 ± 1.6	0.049	6.76 ± 1.92	95.3	33.6
Cupper (mg)	1.23 ± 0.3	1.16 ± 0.2	0.102	1.19 ± 0.30	–	–
Vitamin B1 (mg)	1.05 ± 0.4	0.85 ± 0.3	0.020	0.89 ± 0.34	80.4	23.4
Vitamin B2 (mg)	0.68 ± 0.5	0.52 ± 0.3	0.202	0.55 ± 0.36	93.5	67.3
Vitamin B6 (mg)	0.67 ± 0.28	0.6 ± 0.19	0.317	0.62 ± 0.21	100	82.2
Folate (μg)	91.3 ± 30	84.1 ± 28	0.319	85.5 ± 28.8	100	100
Vitamin C (mg)	35.2 ± 40	38 ± 33	0.748	37.53 ± 34.8	65.4	44.9
Vitamin A (μg)	192.7 ± 146	188.7 ± 185	0.927	189.4 ± 177	96.3	79.4
Vitamin D (μg)	2.87 ± 1.8	2.18 ± 1.6	0.105	2.3 ± 1.72	100	100
Vitamin E (μg)	4.57 ± 1.08	3.88 ± 1.04	0.010	4.02 ± 1.08	100	69.2

^a^ = Energy intake <30 kacl/kg body weight per day; ^b^ = Energy intake <20 kacl/kg body weight per day; ^c^ = Protein intake <1.0 g/kg body weight per day; ^d^ = Protein intake <0.8 g/kg body weight per day; *Independent Sample T test were used to compare the depressed vs non-depressed older adults.

According to Spearman correlation coefficients, Table 4 shows the relationships between the MNA scores and GD scores, age, dietary energy and nutrients, and ADL level, as well as other relationships between other factors. There is a significant inverse association between MNA score and GD score (r = −0.684, p=<0.001). The MNA score is also significantly negatively associated with age. The MNA score, on the other hand, is significantly positively associated with dietary energy, protein, and fiber intake. Dietary energy, protein, and dietary fiber intake were significantly negatively correlated with individuals’ age. Age was considered a significant factor for the MNA score, GD score, ADL, and also dietary energy and nutrient intake (Table 4). The limitation of ADL is also significantly associated with dietary nutrient intake.

**Table 4:** Spearman Correlation between MNA score, Age, GD score, ADL.

Variables/Factors	R	*P*-value
MNA score vs GD score	−0.684	<0.001**
MNA score vs Age	−0.233	0.015*
MNA score vs energy intake	0.303	0.002*
MNA score vs Protein intake	0.203	0.036*
MNA score vs Dietary Fiber intake	0.320	0.001*
Age vs energy intake	−0.198	0.041*
Age vs Protein intake	−0.218	0.024*
Age vs Dietary Fiber intake	−0.221	0.022*
GD score vs Age	0.10	0.302
GD score vs Energy intake	−0.076	0.434
GD score vs Protein intake	−0.057	0.559
GD score vs ADL (0–5, severe limitation to normal)	−0.263	0.006*
ADL (severe limitation to normal) vs Energy intake	0.245	0.011*
ADL (severe limitation to normal) vs Protein intake	0.214	0.027*
ADL (severe limitation to normal) vs Dietary Fiber intake	0.247	0.010*

MNA, Mini Nutritional Assessment; GD, Geriatric Depression; ADL, Activities daily living; *(p < 0.05); ** (p < 0.001).

## 4 Discussion

This study tries to identify the relationship between nutritional status and the level of depression among the older adults living in the community. This research also focused on the risk factors for geriatric malnutrition and depression symptoms. MNA-SF is a screening and assessment tool for malnutrition that can be used in all geriatric settings. The short form of GDS is commonly used to measure depression or the mental health of older persons [23]. The results revealed a high number of older people to be malnourished or at risk of being undernourished at the community level.

The rate of malnourished or at-risk of malnutrition in the current study was almost close to the recent findings from other studies in Bangladesh in a similar setting [6,7]. But the prevalence of malnutrition is significantly lower in this study compared to older adults living in residential aged care facilities in Bangladesh, where they reported the proportion of malnutrition as 33.5% [8]. Earlier studies carried out in Nepal and India also demonstrated a consistent rate of malnutrition [[28], [29], [30]]. But the prevalence of malnutrition in developed countries like Japan, Switzerland, Sweden, France, and South Africa is lower (5.8%), according to data from community-dwelling elderly populations [31]. The high prevalence of malnutrition among older adults in developing countries as compared to developed countries may be due to the unavailability of health care facilities, family support, loneliness, social safety net programs, nutritional guidelines for older adults etc. In this study, the prevalence of malnutrition was lower in males (15.4%) than in females (26.8%). Consistent with our findings, the previous studies from Bangladesh [7], India [30] and Nepal [29] indicated that malnutrition was more prevalent among females compared to their male counterparts. This may be linked to features including the status of women in communities and economic burdens, both of which have an impact on nutrition [32]. Even though older persons without education beyond the primary and secondary levels in this study had a higher percentage of malnutrition or being at risk of it, the difference was not statistically significant. But in the previous study, the difference was found to be significantly higher [7].

In the current study, about 81.5% of the participants showed signs of depression. This is very similar to a study done in Bangladesh [6] in which about 84% of the participants showed a variety of depression symptoms. Like in the previous studies, the depression rate was higher in women as compared to men [33,34]. Like another previous study [35] the current study also found an Inverse correlation between the MNA-SF score and the GD score. Patients with malnutrition had a considerably increased chance of developing depression than those with well-nourished individuals, which was also observed in one of the previous studies [6]. It is unclear whether depression is a cause or a result of malnutrition, but it is obvious that depression has a significant impact on appetites, nutritional practices, and food consumption [35,36]. Depression is linked to alterations in a few neurotransmitters as well as hormonal changes. A diversified, healthier diet with balanced nutrition can lower the chance of depression [37].

Malnutrition is not just linked to depression in the elderly. Previous research on Mexican nursing home residents revealed a high correlation between physical dependence and poor nutritional status [38]. Previous research in Bangladesh by Karim et al. [8] and Ferdous et al. [24] found a similar result. The current study found that the limitation of ADL is significantly associated with dietary nutrient intake. Physical dysfunction has also been identified as a prime cause of reduced food intake, along with other factors such as physiological changes and a lack of nutrition-sensitive care [39]. Additionally, malnutrition among older adults can lead to physical frailty, which might increase depression symptoms [40].

In the current study, we found that the average dietary energy intake of the elderly was 1387 kcal/day, which is similar to a Chinese study where they found 1436.0 kcal/d [16]. This study found that 80.4% of people lacked enough energy, which was almost the same as the previous study by Zhao et al. [16]. Also, 78% of the older people in India who participated did not eat enough calories each day [41]. Like this study’s finding, the prevalence of inadequacy for energy increased with age [16]. Resting energy expenditure (REE) commonly decreases with increasing age, mainly due to decreased fat-free body mass. REE measurements in healthy and ill older adults produced roughly 20 kcal/kg body weight (BW)/day [42]. About 27.1% of the participants in this trial, compared to 16% in a prior study, had very low daily energy consumption (20 kcal/kg body weight/day) [18].

The average protein intake of the current study is in line with a previous study conducted in China, where they found 43.9 g/day [16]. Where they reported that most older adults failed to meet the estimate average requirement (EAR) for protein (76.5%) [16], but the current study observed that most of the participants failed to meet the requirement for protein (76.7%). Adequate dietary protein is significant for maintaining muscle mass and physical performance [43]. In a meta-analysis, older persons living in the community who consumed more protein, 1 g/BWkg/day as opposed to 0.8 g/BWkg/day, had higher mobility and lower limb physical capability [43]. Micronutrient deficiencies are common in this study. More than 90% of the study subjects are deficient in dietary calcium, iron, zinc, vitamin B2, vitamin B6, folate, vitamin A, vitamin D, and vitamin E. Whereas in a previous study, they reported that more than 90% were deficient in vitamin A, vitamin B1, vitamin B2, folate, and calcium [16]. In another study, they found that iron and vitamin D were more deficient in the older subjects [18]. In a recent review, research found that low consumption of proteins, carbohydrates, lipids, dietary fiber, vitamins (D, E, C, and B), and microelements like magnesium, zinc, selenium, or iron have an impact on the development of depression [44]. In the current study, dietary intake of energy, protein, fat, and some of the micronutrients such as zinc, vitamin B1, and vitamin E were significantly lower in depressed older adults than in non-depressed individuals; this finding supports the previous study [44]. The essential amino acid tryptophan is the precursor of the neurotransmitter serotonin, and its deficiency is connected to the pathophysiology of depression [45]. As a result, consuming insufficient protein hinders the production of neurotransmitters.

The study has a few advantages. The study observed a significant inverse association between the MNA-SF and GD scores. The study also explored the fact that energy and protein intake were significantly higher in non-depressed older adults than in depressed participants. The study also observed that malnutrition and depression are both linked to physical impairment. A few limitations of our study must be recognized. Due to the self-reported nature of the MNA-SF, GDS, and physical function examinations, there is a good chance that linguistic nuance as well as social and cultural factors will have an impact on responses. A 24-h food recall was used to figure out how much food was eaten, and the accuracy of the data depends on how well the subjects or caretakers remember. The sample size of the study was small and only included a selected area of Bangladesh. Information related to the oral problem, other socioeconomic factors, and biological parameters were not collected to see other determining factors of malnutrition. The findings of the research offered merely a glimpse of the nutritional state of the elderly population in selected locations in Bangladesh, thus establishing broad conclusions is difficult.

## 5 Conclusion

The study shows that there are a lot of older adults in the community who are malnourished or at risk of being malnourished. The study also shows that the MNA score and the depression score are linked in the opposite way. Malnutrition and depression are linked to other factors, like being unable to move around or do activities of daily living (ADL). Dietary energy and protein intake were lower in the malnourished group than in the well-nourished group. The dietary intakes of energy and most of the nutrients were all below the RDA. To improve the nutritional status of older people, specific nutrition-related intervention programs are recommended. Mental health and other health-related problems should also be emphasized to reduce the risk of malnutrition.

## Ethical consideration

The Daffodil International University Ethics Committee evaluated and approved the study protocol (Ref. No.:/DIU/2019/1004), and which was carried out following the recommendations made by the Declaration of Helsinki. Following an explanation of the survey's objectives to the participants, those who were willing to participate in the study gave their informed consent.

## Author contribution statement

Tasmia Tasnim, Kazi Muhammad Rezaul Karim: Conceived and designed the experiments; Performed the experiments; Analyzed and interpreted the data; Contributed reagents, materials, analysis tools or data; Wrote the paper. Md. Zafar As Sadiq: Performed the experiments; Analyzed and interpreted the data; Wrote the paper.

## Data availability statement

Data will be made available on request.

## Funding

The authors received no financial support for the research, authorship, and publication of this manuscript.

## Declaration of competing interest

The authors declare that they have no known competing financial interests or personal relationships that could have appeared to influence the work reported in this paper.

## References

[1] United Nations (2019). Department of economic and social affairs, population division. World population ageing: highlights (ST/ESA/SER.A/430). https://digitallibrary.un.org/record/3846855?ln=en.

[2] Haque M.M., Uddin A.K.M.M., Naser M.A., H Khan M.Z., K Roy S., Arafat Y. (2014). Health and nutritional status of aged people. CMOSHMC J..

[3] Fávaro-Moreira N.C., Krausch-Hofmann S., Matthys C., Vereecken C., Vanhauwaert E., Declercq A., Bekkering G.E., Duyck J. (2016). Risk factors for malnutrition in older adults: a systematic review of the literature based on longitudinal data. Adv. Nutr. (Bethesda, Md.).

[4] Van Bokhorst-de van der Schueren M.A., Lonterman-Monasch S., de Vries O.J., Danner S.A., Kramer M.H., Muller M. (2013). Prevalence and determinants for malnutrition in geriatric outpatients. Clin. Nutr. (Edinb.).

[5] Chen C.C., Bai Y.Y., Huang G.H., Tang S.T. (2007). Revisiting the concept of malnutrition in older people. J. Clin. Nurs..

[6] Alam M.R., Karmokar S., Reza S., Kabir M.R., Ghosh S., Mamun M. (2021). Geriatric malnutrition and depression: evidence from elderly home care population in Bangladesh. Prev. Med. Rep..

[7] Rahman K., Khalequzzaman M., Khan F.A., Rayna S.E., Samin S., Hasan M., Islam S.S. (2021). Factors associated with the nutritional status of the older population in a selected area of Dhaka, Bangladesh. BMC Geriatr..

[8] Karim K.M.R., Tasnim T., Shams S.D., Zaher M.A., Mamun S. (2022). Mini Nutritional Assessment and physical function of older people in residential aged care facility, Bangladesh. Nutr. Health.

[9] Ahmed T., Haboubi N. (2010). Assessment and management of nutrition in older people and its importance to health. Clin. Interv. Aging.

[10] Ahmadi S.M., Mohammadi M.R., Mostafavi S.A., Keshavarzi S., Kooshesh S.M., Joulaei H., Sarikhani Y., Peimani P., Heydari S.T., Lankarani K.B. (2013). Dependence of the geriatric depression on nutritional status and anthropometric indices in elderly population. Iran. J. Psychiatry.

[11] Krishnan V., Nestler E.J. (2008). The molecular neurobiology of depression. Nature.

[12] Mazumder H., Murshid M.E., Faizah F., Hossain M.M. (2020). Geriatric mental health in Bangladesh: a call for action. Int. Psychogeriatr..

[13] WHO (2016).

[14] Cereda E., Pedrolli C., Klersy C., Bonardi C., Quarleri L., Cappello S., Turri A., Rondanelli M., Caccialanza R. (2016). Nutritional status in older persons according to healthcare setting: a systematic review and meta-analysis of prevalence data using MNA®. Clin. Nutr. (Edinb.).

[15] Cereda E., Veronese N., Caccialanza R. (2018). The final word on nutritional screening and assessment in older persons. Curr. Opin. Clin. Nutr. Metab. Care.

[16] Zhao F., He L., Zhao L., Guo Q., Yu D., Ju L., Fang H. (2021). The status of dietary energy and nutrients intakes among Chinese elderly aged 80 and above: data from the CACDNS 2015. Nutrients.

[17] Iuliano S., Olden A., Woods J. (2013). Meeting the nutritional needs of elderly residents in aged-care: are we doing enough?. J. Nutr. Health Aging.

[18] Engelheart S., Akner G. (2015). Dietary intake of energy, nutrients and water in elderly people living at home or in nursing home. J. Nutr. Health Aging.

[19] Islam M.Z., Disu T.R., Farjana S. (2021). Malnutrition and other risk factors of geriatric depression: a community-based comparative cross-sectional study in older adults in rural Bangladesh. BMC Geriatr..

[20] Rubenstein L., Harker J., Salva A., Guigoz Y., Vellas B. (2001). Screening for undernutrition in geriatric practice: developing the short-form mini-nutritional assessment (MNA-SF). J. Gerontol. Biol. Med. Sci..

[21] Sarikaya D., Halil M., Kuyumcu M.E. (2015). Mini nutritional assessment test long and short form are valid screening tools in Turkish older adults. Arch. Gerontol. Geriatr..

[22] Sheikh J.I., Yesavage J.A. (1986). Geriatric depression scale (GDS): recent evidence and development of a shorter version. Clin. Gerontol.: J Aging Mntl Health.

[23] Greenberg S.A. (2007). How to try this: the geriatric depression scale: short form. Am. J. Nurs..

[24] Ferdous T., Cederholm T., Razzaque A., Wahlin A., Nahar Kabir Z. (2009). Nutritional status and self-reported and performance-based evaluation of physical function of elderly persons in rural Bangladesh. Scand. J. Publ. Health.

[25] Shahen N., Rahim A.T.M.A., Mohiduzzaman M., Banu C.P., Bari M.L., Tukun A.B., Mannan M.A., Bhattacharjee L., Stadlmayr B. (2013).

[26] Volkert D., Beck A.M., Cederholm T., Cruz-Jentoft A., Goisser S., Hooper L. (2019). ESPEN guideline on clinical nutrition and hydration in geriatrics. Clin. Nutr..

[27] (2001). FAO/WHO Human Vitamin and Mineral Requirements, Report of a Joint FAO/WHO Expert Consultation, Bangkok.

[28] Tamang M., Yadav U.N., Hosseinzadeh H., Kafle B., Paudel G., Khatiwada S., Sekaran V.C. (2019). Nutritional assessment and factors associated with malnutrition among the elderly population of Nepal: a cross-sectional study. BMC Res. Notes.

[29] Ghimire S., Baral B.K., Callahan K. (2017). Nutritional assessment of community-dwelling older adults in rural Nepal. PLoS One.

[30] Lahiri S., Biswas A., Santra S., Lahiri S. (2015). Assessment of nutritional status among elderly population in a rural area of West Bengal, India. Int. J. Med. Sci. Publ. Health.

[31] Kaiser M.J., Bauer J.M., Rämsch C., Uter W., Guigoz Y., Cederholm T., Thomas D.R., Anthony P.S., Charlton K.E., Maggio M., Tsai A.C., Vellas B., C Sieber C., Mini Nutritional Assessment International Group (2010). Frequency of malnutrition in older adults: a multinational perspective using the mini nutritional assessment. J. Am. Geriatr. Soc..

[32] Agarwalla R., Saikia A.M., Baruah R. (2015). Assessment of the nutritional status of the elderly and its correlates. J Family Community Med.

[33] Vafaei Z., Mokhtari H., Sadooghi Z., Meamar R., Chitsaz A., Moeini M. (2013). Malnutrition is associated with depression in rural elderly population. J. Res. Med. Sci..

[34] Chrzastek Z., Guligowska A., Soltysik B., Piglowska M., Borowiak E., Kostka J., Kostka T. (2021). Association of lower nutritional status and education level with the severity of depression symptoms in older adults—a cross sectional survey. Nutrients.

[35] Velázquez-Alva M.C., Irigoyen-Camacho M.E., Cabrer-Rosales M.F., Lazarevich I., Arrieta-Cruz I., Gutiérrez-Juárez R., Zepeda-Zepeda M.A. (2020). Prevalence of malnutrition and depression in older adults living in nursing homes in Mexico city. Nutrients.

[36] Al-Rasheed R., Alrasheedi R., Al Johani R., Alrashidi H., Almaimany B., Alshalawi B., Kelantan A., Banjar G., Alzaher A., Alqadheb A. (2018). Malnutrition in elderly and its relation to depression. IJCMPH.

[37] Li Y., Lv M.R., Wei Y.J., Sun L., Zhang J.X., Zhang H.G., Li B. (2017). Dietary patterns and depression risk: a meta-analysis. Psychiatr. Res..

[38] Velázquez-Alva M.C., Irigoyen-Camacho M.E., Zepeda-Zepda M.A., Lazarevich I., Arrieta-Cruz I., D’Hyver C. (2020). Sarcopenia, nutritional status and type 2 diabetes mellitus: a cross-sectional study in a group of Mexican women residing in a nursing home. Nutr. Diet..

[39] Tsuji T., Yamamoto K., Yamasaki K. (2019). Lower dietary variety is a relevant factor for malnutrition in older Japanese home-care recipients: a cross-sectional study. BMC Geriatr..

[40] Bickford D., Morin R.T., Woodworth C., Verduzco E., Khan M., Burns E., Nelson J.C., Mackin R.S. (2021). The relationship of frailty and disability with suicidal ideation in late life depression. Aging Ment. Health.

[41] Gupta A., Khenduja P., Pandey R.M., Sati H.C., Sofi N.Y., Kapil U. (2017). Dietary intake of minerals, vitamins, and trace elements among geriatric population in India. Biol. Trace Elem. Res..

[42] Gaillard C., Alix E., Salle A., Berrut G., Ritz P. (2008). A practical approach to estimate resting energy expenditure in frail elderly people. J. Nutr. Health Aging.

[43] Coelho-Júnior H.J., Milano-Teixeira L., Rodrigues B., Bacurau R., Marzetti E., Uchida M. (2018). Relative protein intake and physical function in older adults: a systematic review and meta-analysis of observational studies. Nutrients.

[44] Chrzastek Z., Guligowska A., Sobczuk P., Kostka T. (2023). Dietary factors and risk of developing depression and severity of its symptoms in older adults–a narrative review of current knowledge. Nutrition.

[45] Kraus C., Castrén E., Kasper S., Lanzenberger R. (2017). Serotonin and neuroplasticity–links between molecular, functional and structural pathophysiology in depression. Neurosci. Biobehav. Rev..

